# Steroid diversification by multicomponent reactions

**DOI:** 10.3762/bjoc.15.121

**Published:** 2019-06-06

**Authors:** Leslie Reguera, Cecilia I Attorresi, Javier A Ramírez, Daniel G Rivera

**Affiliations:** 1 Center for Natural Product Research, Faculty of Chemistry, University of Havana, Zapata y G, Havana 10400, Cuba; 2 Departamento de Química Orgánica, Facultad de Ciencias Exactas y Naturales, Universidad de Buenos Aires. Ciudad Universitaria, Ciudad Autónoma de Buenos Aires, C1428EGA, Argentina; 3 CONICET – Universidad de Buenos Aires. Unidad de Microanálisis y Métodos Físicos Aplicados a Química Orgánica (UMYMFOR). Ciudad Universitaria, Ciudad Autónoma de Buenos Aires, C1428EGA, Argentina

**Keywords:** conjugation, heterocycles, macrocycles, multicomponent reactions, steroids

## Abstract

Reports on structural diversification of steroids by means of multicomponent reactions (MCRs) have significantly increased over the last decade. This review covers the most relevant strategies dealing with the use of steroidal substrates in MCRs, including the synthesis of steroidal heterocycles and macrocycles as well as the conjugation of steroids to amino acids, peptides and carbohydrates. We demonstrate that steroids are available with almost all types of MCR reactive functionalities, e.g., carbonyl, carboxylic acid, alkyne, amine, isocyanide, boronic acid, etc., and that steroids are suitable starting materials for relevant MCRs such as those based on imine and isocyanide. The focus is mainly posed on proving the amenability of MCRs for the diversity-oriented derivatization of naturally occurring steroids and the construction of complex steroid-based platforms for drug discovery, chemical biology and supramolecular chemistry applications.

## Review

### 1 Introduction

**Figure 1:**
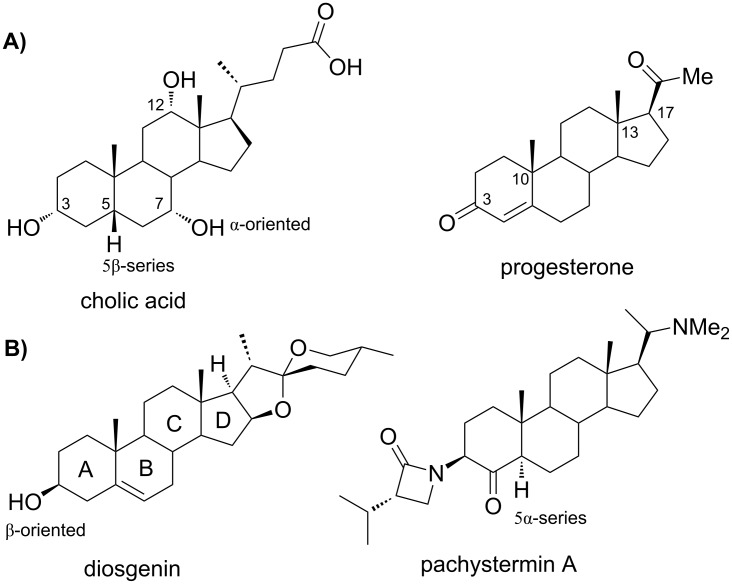
Structures of natural steroids of A) animal and B) plant origin.

The utilization of multicomponent reactions (MCRs) [1] for the derivatization of biomolecules has continuously grown over the last years. These diversity-oriented and complexity-generating processes [2–3] have proven success in peptide ligation [4] and macrocyclization [5–6], protein glycoconjugation [7], lipidation of peptides [8] and glycosides [9], and carbohydrate modification [10]. A special class of lipidic biomolecules are the steroids, which can be either totally lipophilic (e.g., cholesterol) or amphipathic when possessing both polar and nonpolar groups (e.g., cholic acid). As shown in Figure 1, members of this family can be found in both plant and animal kingdoms, where they exert an amazing array of cellular functions such as structural and hormonal ones. All steroids are based on a common skeleton containing three fused six-membered rings and one five membered ring. This fused-ring system provides a readily available source of rigidity and chirality, whose substituents can be oriented either towards the α- or the β-face. Steroids feature a polyprenyl nature, which is particularly evident in the side chain, and have additional structural elements such as the methyl groups at positions 10 and 13, always β-oriented. Other positions of the steroidal nucleus of frequent natural functionalization are C-3 (usually bearing a hydroxy group) and C-17, this latter holding the aliphatic side chain but also often having oxygenated functions [11]. Similarly, rings B and C can be naturally functionalized with a varied set of groups including hydroxy, carbonyl and olefins, which along with those present in rings A and D and the side chain are the basis of steroid biological functions. Such oxygenated and alkene groups represent the entrance door for subsequent synthetic modifications, including the incorporation of MCR reactive functionalities.

In the last two decades, MCRs have emerged as effective tools for the rapid derivatization of steroidal skeletons in the pursuit of medicinal and supramolecular chemistry applications. Despite some review articles have described selected examples of MCRs used to modify and to macrocyclize steroidal compounds [12–13], to our knowledge there is no review exclusively dedicated to covering the applications of MCRs with steroids. Herein, we provide a comprehensive review describing – to our understanding – the most important inputs of the utilization of MCRs in the structural diversification of steroids. The review is divided in different aspects of steroid chemistry, such as modification of the side chain and the steroidal nucleus, the assembly of nitrogen-heterocycles fused to the steroid-ring system, the steroid conjugation and macrocyclization, all relying on MCRs.

### 2 Modification of the steroidal nucleus and the side chain

#### 2.1 Isocyanide-based MCRs

**Scheme 1:**
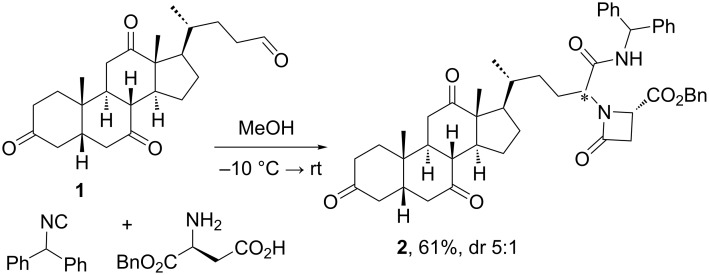
Synthesis of a steroidal β-lactam by Ugi reaction of a cholanic aldehyde [14].

**2.1.1 Steroids as carbonyl component:** One of the first steroid derivatization methods using MCRs was reported by the Ugi laboratory in 1995 (Scheme 1) [14]. The occurrence of pachystermin A, an unusual natural steroid isolated from the boxaceous plant *Pachysandra terminalis* and having a β-lactam moiety in ring A, inspired these authors to obtain a steroidal β-lactam, albeit functionalized in the side chain. The one-pot synthesis of the β-lactam steroid was achieved via the Ugi 3-component-4-center reaction using the dehydrocholic aldehyde **1** as carbonyl component. This variation of the Ugi reaction including a β-amino acid component allows the formation of the 4-membered ring of the β-lactam moiety, which is difficult to obtain through other traditional methods like the cyclization of acyclic precursors because of both the Baeyer strain of the newly formed ring and conformational effects [15]. However, the readily formation of the 4-membered ring by this MCR is due to the ring contraction of the 7-membered ring cyclic intermediate by transannular acyl transfer, which leads to the β-lactam derivative. Despite the chiral nature of the steroidal substrate, no significant stereoselective induction was observed, probably because the 7-membered ring intermediate was far away from the chiral steroidal nucleus.

**Scheme 2:**
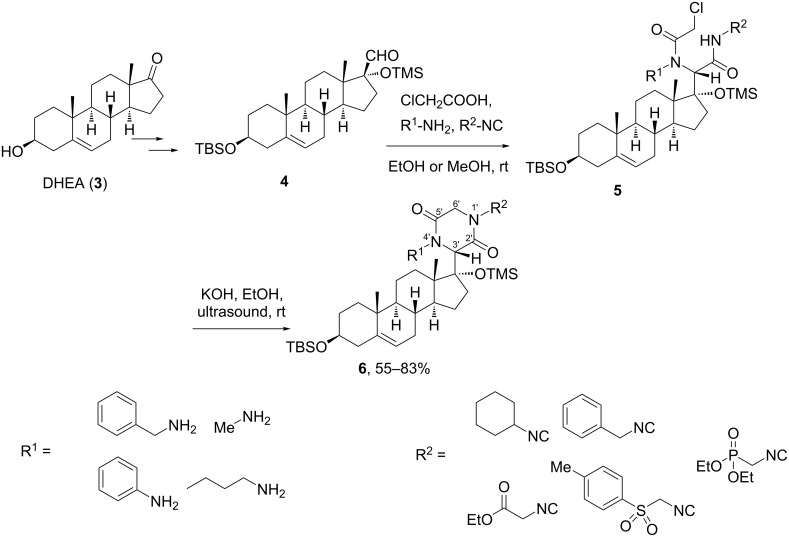
Synthetic route to steroidal 2,5-diketopiperazines based on a diastereoselective Ugi-4CR with an androstanic aldehyde derived from dehydroepiandrosterone (DHEA) [16].

A better stereoselectivity was reported by Bruttomesso et al. in their work on the Ugi four-component reaction (Ugi-4CR) with a steroidal aldehyde, in which the carbonyl group was directly linked at the steroidal framework [16]. As shown in Scheme 2, the authors found a high diastereoselectivity in the Ugi-4CR conducted with a carbonyl linked at C-17, a sterically crowed position very close to the axial methyl group placed of C-13. This step was implemented as part of the synthetic route toward steroidal 2,5-diketopiperazine **6**, based on a strategy previously proposed by Marcaccini and co-workers [17]. Here, the route included the Ugi-4CR reaction of varied amines and isocyanides with the androstanic aldehyde **4** and chloroacetic acid followed by a post-cyclization reaction by treating the Ugi products **5** with ethanolic KOH under ultrasonication. This protocol provided a small library of steroidal 2,5-diketopiperazines **6** obtained as a single diastereomer, thus proving the high diastereoselection of the initial Ugi-4CR with the steroidal carbonyl substrate. In the 2,5-diketopiperazine ring, the configuration of the new asymmetric center C-3’ was *S*, a result confirmed by NOESY experiments.

In comparison with the work described above, it can be suggested that in the case of the 2,5-diketopiperazine synthesis, the distance of the carbonyl moiety to the steroidal nucleus is determinant for the diastereoselection of the Ugi-type reaction. Thus, when the carbonyl is far away from the rigid chiral steroid, the Ugi reaction shows no significant diastereoselectivity, while the carbonyl component directly linked to the bulky steroidal nucleus – especially in α-position of a stereocenter – provides a very good diastereocontrol in the MCR.

**Scheme 3:**
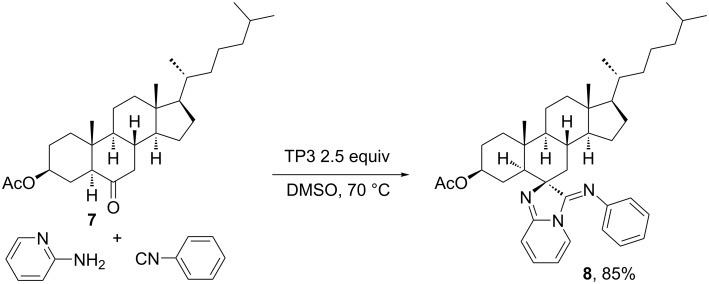
Multicomponent synthesis of a heterocycle–steroid hybrid using a ketosteroid as carbonyl component [18].

Dar et al*.* [18] reported another example of a diastereoselective MCR between a 6-ketosteroid, 2-aminopyridines and an isocyanide, using propylphosphonic acid anhydride (TP3) as catalyst to afford a steroidal derivative with an imidazole-pyridine moiety attached to ring B. This moiety is present in several compounds displaying biological activities such as antimicrobial, antiviral and anti-inflammatory, among others. Scheme 3 exemplifies the reaction of cholestan-6-one **7** with 2-aminopyridine and phenyl isocyanide to afford the heterocycle–steroid hybrid **8** in good yield and diastereoselectivity, whereas different ketosteroids could be used as starting materials, thus proving the versatility of this reaction. Also, this group extended the reaction scope to different 2-aminopyridines with electron-donating groups in *para* and *meta* positions, and to benzyl and other alkyl isocyanides. The proposed mechanism follows the first step of the Ugi-4CR reaction, i.e., the formation of the imine between the ketosteroid and the amine group of the aminopyridine derivative. Then, the imine suffers the nucleophilic attack of the isocyanide generating a nitrilium intermediate. Differently from the Ugi-4CR, the final step is the cyclization between the pyridine N atom and the isocyanide. TP3 plays the catalytic role by acidic activation of the ketone to favor the imine formation.

**Scheme 4:**
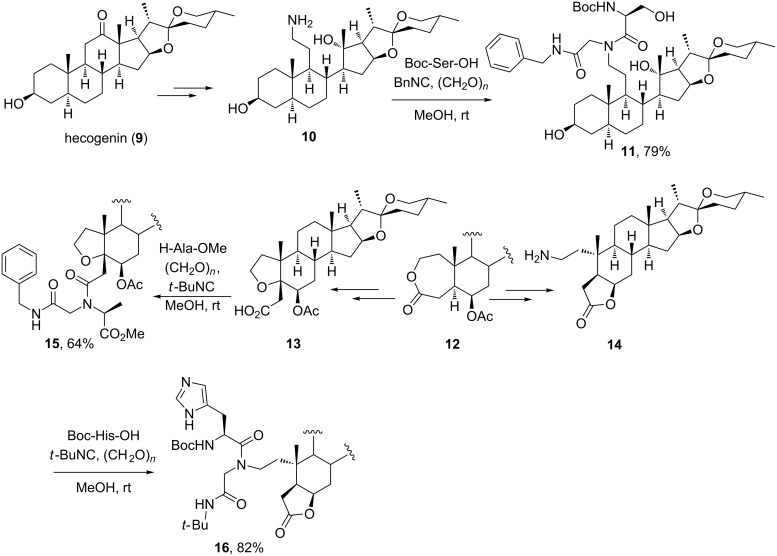
Synthesis of peptidomimetic–steroid hybrids using the Ugi-4CR with spirostanic amines and carboxylic acids [19].

**2.1.2 Steroids as amine and carboxylic acid components:** Several groups have employed steroidal substrates as amino and carboxylic acid components of the Ugi-4CR, which is indeed one of the MCRs of major incidence in the field of steroid chemistry. Rivera and co-workers were the first to employ this 4CR for the conjugation of amino acids to steroids [19] as part of a general program for the synthesis of peptidomimetic–steroid hybrids [20–21]. As shown in Scheme 4, the authors developed methods for the functionalization of spirostanic steroids and their further conjugation to amino acids by means of the Ugi-4CR. Thus, seco-steroidal amine **10** – derived from the steroidal sapogenin hecogenin (**9**) – was ligated to Boc-protected serine in presence of paraformaldehyde and benzyl isocyanide to form hybrid **11** in good yield. Similarly, the spirostanic lactone **12** was transformed into spirostanic acid **13** and amine **14**, which were next ligated to alanine methyl ester and Boc-histidine leading to the amino acid–steroid conjugates **15** and **16**, respectively. This approach was also employed for the ligation of amino acids to spirostanic seco-steroids at ring B and for the simultaneous incorporation of two amino acid residues [19].

**Scheme 5:**
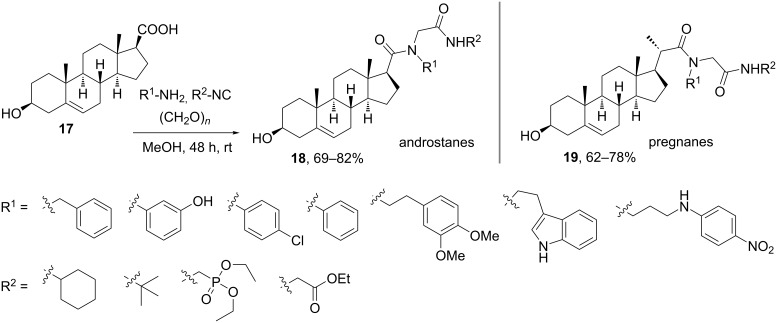
Synthesis of azasteroids using the Ugi-4CR with androstanic and pregnanic carboxylic acids [22].

Ramírez and co-workers have extended the application of the Ugi-4CR to the diversity-oriented functionalization of androstanic and preganinc steroids using a carboxylic acid group at the side chain [22–23]. As shown in Scheme 5, the implementation of the Ugi-4CR with a variety of amines and isocyanides – keeping formaldehyde as the oxocomponent – enabled the generation of azasteroid libraries based on androstanic (**18**) and pregnanic (**19**) skeletons. These azasteroids, in which one or more nitrogen atoms are present in the side chain, were designed as promising compounds since such classes of antifungal steroids were known in the literature.

Some of the resulting azasteroids showed inhibitory effects on the growth of fungi such *Fusarium lateritium* (causal agent of chlorotic leaf distortion in sweet potato) and *Fusarium virguliforme* (causal agent of sudden death syndrome in soy bean), without exerting in vitro toxicity on mammalian cells. Another interesting result came out from the comparison of the biological activity between homologous pairs **18** and **19** [22]. For example, the pair of androstanic and pregnanic derivatives incorporating R^1^ = Phe and R^2^ = *t*-Bu showed significant activity against both fungi tested, but the compounds derived from benzylamine (i.e., R^1^ = Bn) did exhibit either a low or null biological activity for the fungi. This could indicate that the antifungal properties of such azasteroids very much depend on the phenyl moieties attached to the side chain. This type of biological comparison could be established because of the versatility of the synthetic strategy based on the MCR tool.

**Figure 2:**
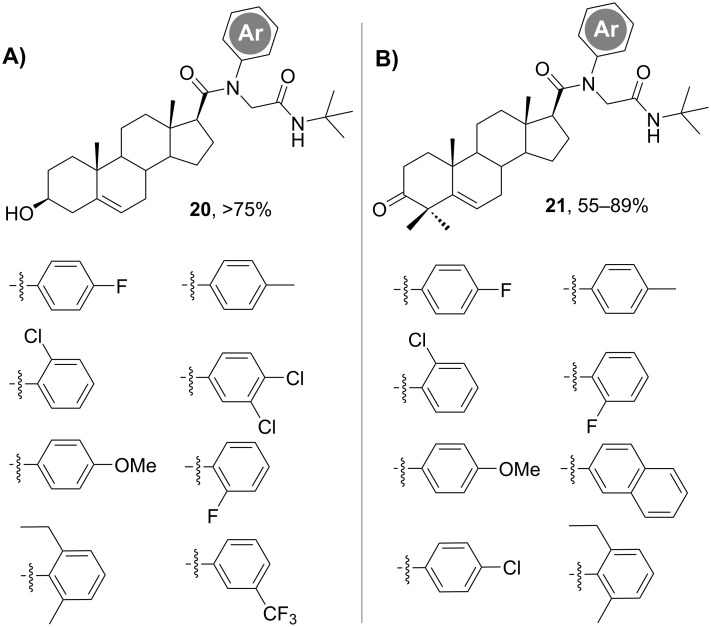
Ugi-4CR-derived library of androstanic azasteroids with diverse substitution patterns at the phenyl ring (amine component) [23].

On the other hand, the family of compound class **18** was evaluated for the inhibition of the viral multiplication and for their implication in intracellular localization of viral glycoproteins [23]. Two compounds showed antiviral effects against herpes simplex virus 1 (HSV-1, KOS strain) and vesicular stomatitis virus (VSV). The key structural characteristics of both compounds are the presence of a phenyl moiety as R^1^ and a *t*-Bu group at the terminal amide. Thus, using the same synthetic strategy as shown in Scheme 5, Dávola et al. [23] synthetized a family of new Ugi reaction-derived androstanic azasteroids with a much more diverse substitution pattern at the phenyl moiety (Figure 2A).

The small library of compounds enabled a better understanding of the structural factors determining the antiviral activity versus the cytotoxic one, with the major effect found for substituents at the *para-*position [23]. Eventually, this work allowed the design of new compounds with antiviral activity and reduced cytotoxic effects by changing the type and position of the substituent. Such small structural changes could be implemented in a rapid manner because of the easy setup of the MCR approach.

To get a deeper insight into the biological effect of Ugi-derived steroids, additional libraries were synthesized by Ramírez, Barquero and co-workers following the concept of varying the amine component and using the steroid as carboxylic acid [24–25]. For example, based on the structure of 4,4-dimethylsterols – compounds that are involved in specific physiological processes – this group used the Ugi-4CR chemistry [24] for the subsequent diversification of the androstanic family of azasteroids, again focusing on the installation of substituted phenyl groups (derived from the amine component) that gave such good results in their previous work [22–23]. The library of androstanic derivatives **21** (Figure 2B) and their reduced analogues bearing the 3β-hydroxy group were biologically tested showing antifungal activity against *Fusarium virguliforme* and *Fusarium solani*. The generation of this compound library suffered from the formation of the Passerini reaction byproducts – likely due to the poor reactivity of the substituted anilines as amino components of the Ugi-4CR. Nonetheless, the Passerini products were also evaluated and provided important information of the structure–activity relationship [24]. An additional library was built by performing the Ugi-4CR at a carboxylic acid placed at C-16 [25], a position never before derivatized by MCRs as its substitution is less common in natural steroids. The authors extended the studies of the C-16 Ugi-derivatized steroids as antiviral agents and found compounds with inhibitory effects against HSV-1 spread (on the wild type and on the acyclovir-resistant strains), as they interfered with late steps in the viral replication cycle [25].

**Scheme 6:**
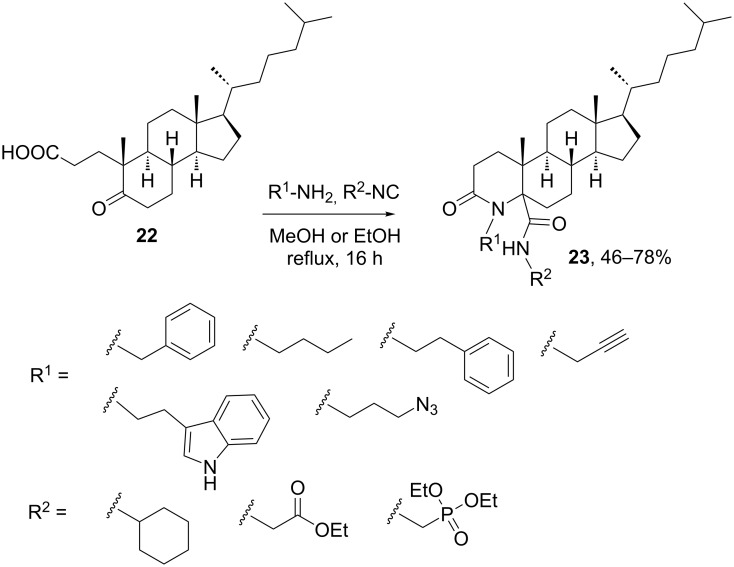
Synthesis of 4-azacholestanes by an intramolecular Ugi-4C-3R [26].

An interesting feature of the Ugi reactions shown with ketosteroids having additional carboxylic and aldehyde groups (see Scheme 1 and Figure 2B) is the lack of side reaction at the ketone functionality. This can be explained by the higher reactivity of the aldehyde functionalities upon imine formation with both aliphatic and aromatic amines. However, steroidal ketones can also participate in Ugi reactions if no additional, more reactive carbonyl components are present. As shown in Scheme 6, this has been demonstrated by Alonso et. al. [26] with the development of a multicomponent approach to obtain 4-azasteroids using an intramolecular Ugi-4CR between a bifunctional steroidal ketoacid and different amines and isocyanides. Because of the poor reactivity of the steroidal ketone, the Ugi-4CR had to be conducted at reflux to obtain a good yield of Ugi-derived steroids, albeit the procedure generated a pair of diastereomers (epimeric at C-5) in almost equal amount. The stereoselectivity of the intramolecular MCR increased when a bulky isonitrile was used, e.g., diethyl phosphonate isocyanide. The configuration of C-5 in each stereoisomer was established using NOESY experiments and theoretical models.

The main limitation of this approach was the impossibility to incorporate both aromatic and sterically hindered amines (e.g., *tert*-butylamine). Besides the reports of Rivera’s and Ramírez’s groups on Ugi-4CRs with spirostanic, androstanic, pregnanic and cholestanic carboxylic acids, later on Chowdhury and co-workers published a similar strategy for the multicomponent derivatization of cholestanes using microwave assisted Ugi-4CR [27].

**Scheme 7:**
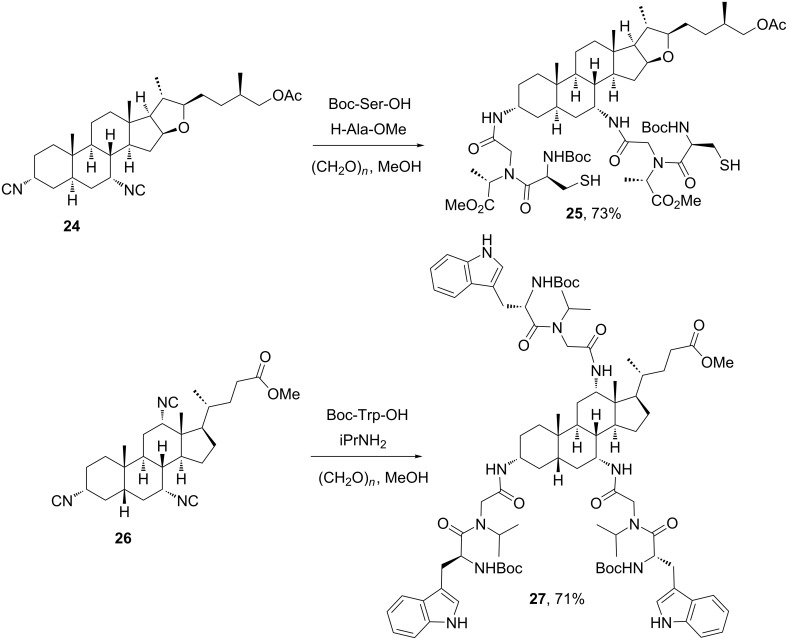
Synthesis of amino acid–steroid hybrid by multiple Ugi-4CR using steroidal isocyanides [29].

**2.1.3 Steroids as the isocyanide component**: To our knowledge, the first synthesis and application in MCRs of steroidal isocyanides was reported by Wessjohann and co-workers [28] during their work on multicomponent macrocyclizations with steroidal building blocks. After that, this group focused on the synthesis of steroidal podands using multiple MCRs for the installation of peptidomimetic chains at different positions of the steroidal skeleton, which enabled rigidifying the pendant peptide chains [29]. Scheme 7 depicts two selected examples of this work, showing the possibility of incorporating isocyanide groups at furostanic and cholanic steroids and their subsequent utilization in the Ugi-4CR with monoprotected amino acids (used either as amino or carboxylic acid components) and formaldehyde as fixed oxo component. Amazingly, the Ugi-4CR of steroidal triisocyanide **26** with three equivalents of Boc-tryptophan allowed the formation of twelve covalent bonds in one-pot, leading to a remarkably complex amino acid–steroid hybrid **27** in very good yield [29].

**Scheme 8:**
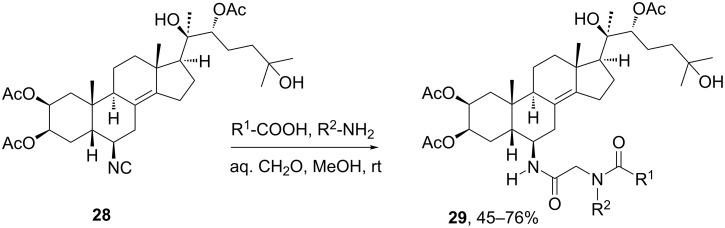
Synthesis of ecdysteroid derivatives by Ugi-4CR using a steroidal isocyanide [30].

After the pioneering work of Wessjohann and co-workers, other groups relied on the functionalization of steroids with isocyanide groups for their subsequent derivatization by isocyanide-based MCRs. Thus, Lesma et al*.* [30] reported an interesting example of generation of an Ugi product at ring B using a steroidal isocyanide featuring an insect hormone ecdysteroid structure. As shown in Scheme 8, a set of Ugi-4CR-derived ecdysteroids **29** was produced by variation of the amine and carboxylic acid components, keeping formaldehyde as oxo component to obtain a single stereoisomer. The library was also integrated by the Ugi-4CR products derived from the 6β-ecdystereoidal amine (also used as precursor of isocyanide **28**), while all compounds were evaluated as antiproliferative agents against T-leukemia cell line.

**Scheme 9:**
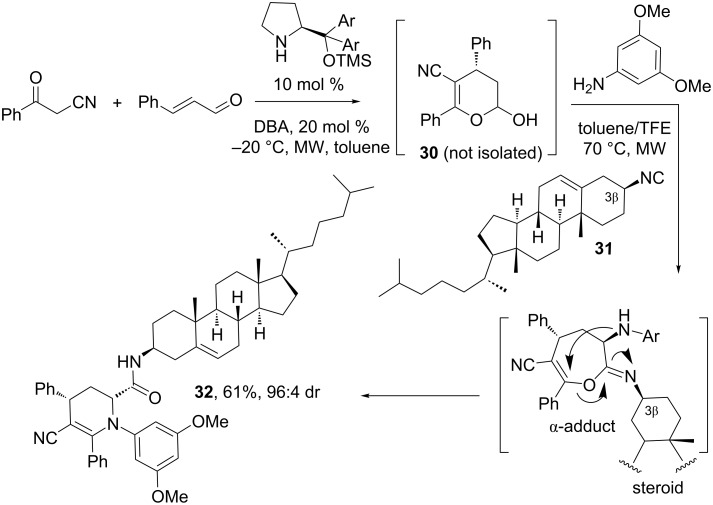
Stereoselective multicomponent synthesis of a steroid–tetrahydropyridine hybrid using a chiral bifunctional substrate and a cholestanic isocyanide. DBA: 3,5-dinitrobenzoic acid [31].

A very recent example of a highly diastereoselective Ugi-type MCR was reported by Rivera, Paixão and co-workers using a steroidal isocyanide [31]. As shown in Scheme 9, the procedure comprised the organocatalytic asymmetric synthesis of the chiral bifunctional substrate **30** bearing an masked aldehyde and an enol functionality, which was subsequently reacted with isocyanide **31** – derived from cholesterol – and 3,5-dimethoxyaniline leading to the steroid–tetrahydropyridine hybrid **32** in good yield and excellent diastereoselectivity. As previously proven by the authors in their synthetic program on organocatalytic multicomponent approaches [32] not only the chiral steroidal isocyanide but also the bifunctional component **30**, used in enantiomerically enriched form, plays a crucial role in the high stereoselection.

The mechanistic insights of this class of MCR were disclosed in the original publication [31]. In short, the organocatalytic conjugated addition of benzoylacetonitrile to cinnamaldehyde generates the hemiacetal **30**, which next initiates the multicomponent sequence upon condensation with the aniline and formation of the imine, eventually occurring as a stable cyclic aminal. The attack of the steroidal isocyanide **31** to the imine (or aminal) leads to the stereoselective formation of the α-adduct intermediates, which rearranges to the final tetrahydropyridine ring **32**.

Besides the use of steroids as oxo, amine, carboxylic acid and isocyanide components, there is a very early report by Dumestre et al. [33] describing the participation of a nitrosteroid and an acylating agent in a novel isocyanide-based MCR. Thus, the reaction of a nitrosteroid – derived from 16-dehydropregnenalone – with acetic anhydride and *tert*-butyl isocyanide furnished an acetyl α-oximinoamide moiety at the steroidal side chain. Albeit this reaction did not find later synthetic applications, it encompasses a nice example of the diversity of MCRs that can be applied to diversify steroidal skeletons.

#### 2.2 Miscellaneous MCRs for steroid derivatization

**Scheme 10:**
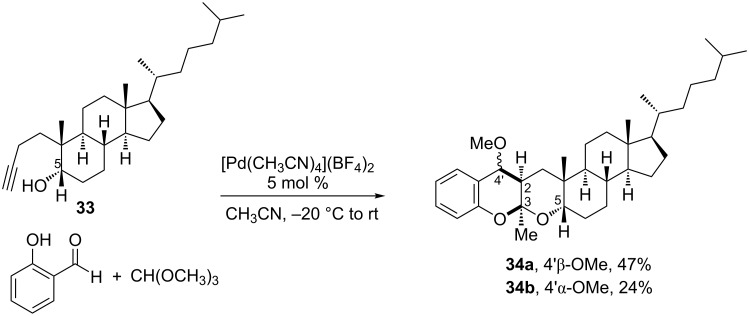
Pd(II)-catalyzed three-component reaction with an alkynyl seco-cholestane [34].

Despite isocyanide-based MCRs have been the main classes of multicomponent transformations used in steroid modification, in the last years various MCRs have emerged as suitable procedures for the diversification of this family of biomolecules. A remarkable example is the report of Iglesias-Arteaga and co-workers [34] on the application of the Pd(II)-catalyzed three-component reaction (3CR) developed by Barluenga et al*.* [35] to steroids. The original Barluenga’s 3CR comprises the reaction of salicylaldehyde with an alkyl orthoformate and 4-pentyn-1-ol to obtain a 4-alkylchroman spiroketal as a single diastereomer. However, as shown in Scheme 10, the employment of alkynyl-4,5-secocholestan-5-ol **33** in such a metal-catalyzed MCR led to the steroidal chroman-ketal **34** as a mixture of epimers at the center bearing the methoxy group, instead of the expected chroman spiroketal. The authors proposed the initial formation of a 6-membered enol ether, which may subsequently undergo either a stepwise Pd(II)-catalyzed cascade process or a [4 + 2] cycloaddition reaction, both providing the *cis* stereochemistry at C-2 and C-3. When the reaction was carried out with the epimer of substrate **33** (having the 5β-hydroxy group), the *cis* stereochemistry was also achieved at the newly formed C-2 and C-3, but with the tetrahydropyrane ring having α orientation. A mixture of epimers at position 4' was also obtained when using the C-5 epimer of substrate **33**.

**Scheme 11:**
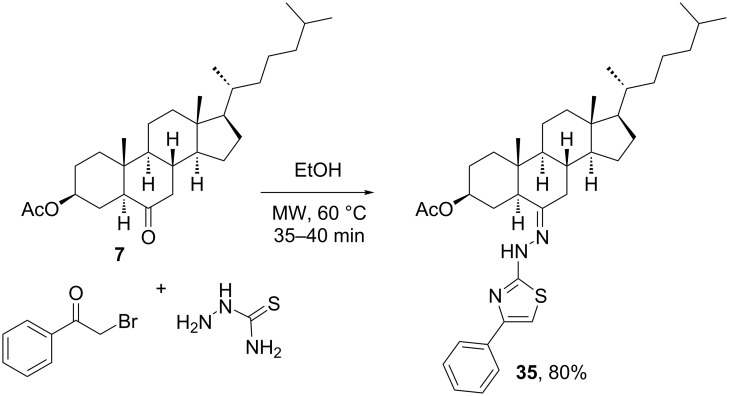
Multicomponent synthesis of steroid–thiazole hybrids from a steroidal ketone [36].

Asif et al. [36] developed another 3CR for the synthesis of steroidal thiazole derivatives. As shown in Scheme 11, cholestanic ketone **7** was reacted with a thiosemicarbazide and 2-bromo-1-phenylethan-1-one under microwave irradiation to form the steroid–thiazole hybrid **35** in very good yield. As previously mentioned, due to the poor reactivity of steroidal ketones and their imine derivatives, most MCRs with ketosteroids described in the literature required a high temperature set by classic heating or microwave irradiation.

**Scheme 12:**
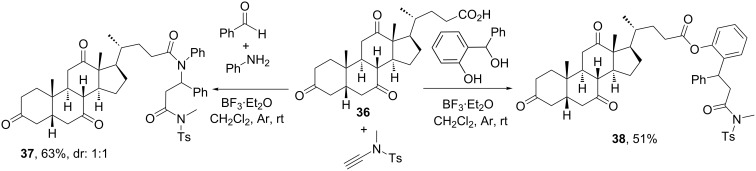
Synthesis of cholanic *pseudo*-peptide derivatives by novel MCRs based on the reactivity of ynamide [37–38].

Using cholic acid as carboxylic acid component, Cui and co-workers [37] developed a novel MCR resembling the Ugi-4CR, but relying on the reactivity of ynamides as surrogates of the isocyanide component. Ynamides are alkynes with a carbon–carbon triple bond attached to a nitrogen atom that gives them both nucleophilic and electrophilic properties. This dual reactivity is similar to that shown by isocyanides, as they may react with iminium ions and carboxylates as isocyanides do. However, in comparison with the typical Ugi-4CR where the isocyanide component acts as a one-carbon center, in this reaction the ynamide compound has a two-carbon center role. As shown in Scheme 12, the reaction of dehydrocholic acid (**36**), an amine and an aldehyde with the key ynamide component provided a new type of *pseudo*-peptidic steroid **37**, obtained as a 1:1 mixture of diastereomers. Besides steroids, the authors proved that a wide variety of components participate in this reaction, unfortunately always rendering mixtures of diastereomers when pro-chiral aldehydes were employed [37]. Also differently from the Ugi-4CR, this MCR requires the use of a Lewis acid like BF_3_·Et_2_O for a suitable activation of the imine.

Based on the same idea of exploiting the reactivity of ynamides, Cui’s group developed a 3CR wherein an *o*-hydroxy-benzhydryl alcohol was used in place of the aldehyde or imine components upon reaction with ynamide and a carboxylic acid [38]. Scheme 12 depicts the implementation of this 3CR with dehydrocholic acid **36** to furnish steroid derivative **38** functionalized at the side chain. This reaction incorporated an ester group at the position where the analogous 4CR created a tertiary amide, because of the similarities between the Passerini and Ugi reactions.

### 3 Synthesis of steroid-fused heterocycles

The modification of the steroidal nucleus by attaching a heterocycle to this hydrophobic scaffold has been traditionally used as an effective way to modulate the biological activity of these biomolecules. In this regard, pentacyclic steroids are a class of pharmacologically relevant steroid derivatives in which the steroid-ring system has a fused heterocyclic or carbocyclic ring. The fifth ring is typically fused to the steroid on ring A, B or D and some times more than one ring is fused, thus generating hexacyclic or heptacyclic steroids. To our knowledge, there are no reports of fused heterocyclic or carbocyclic rings on the ring C of the steroid. In this section, we discuss different reports on the synthesis of steroid-fused heterocycle using MCRs.

Wang et al. [39] developed a synthetic route based on the Biginelli reaction for the preparation of steroidal derivatives with a pyrimidine moiety fused to ring D. This heterocycle moiety appears in different biologically active steroids fused to ring D, and previous non-MCR methods had been reported for the creation of libraries of such hybrid compounds [40]. The Biginelli reaction reported in 1893 comprises the acid–catalyzed condensation of ethyl acetoacetate, benzaldehyde and urea to generate 3,4-dihydropyrimidin-2(1*H*)-one [41]. From that time, the reaction was extended to Lewis-acid catalysis and the use of other solvents such as methanol or aprotic solvents such as THF, dioxane, acetonitrile, etc. Because the reaction rate is slow at room temperature, it needs activation by heating or other non-traditional methods such as ultrasound, microwave, IR irradiation and photochemical irradiation, as it will be exemplified below.

**Scheme 13:**
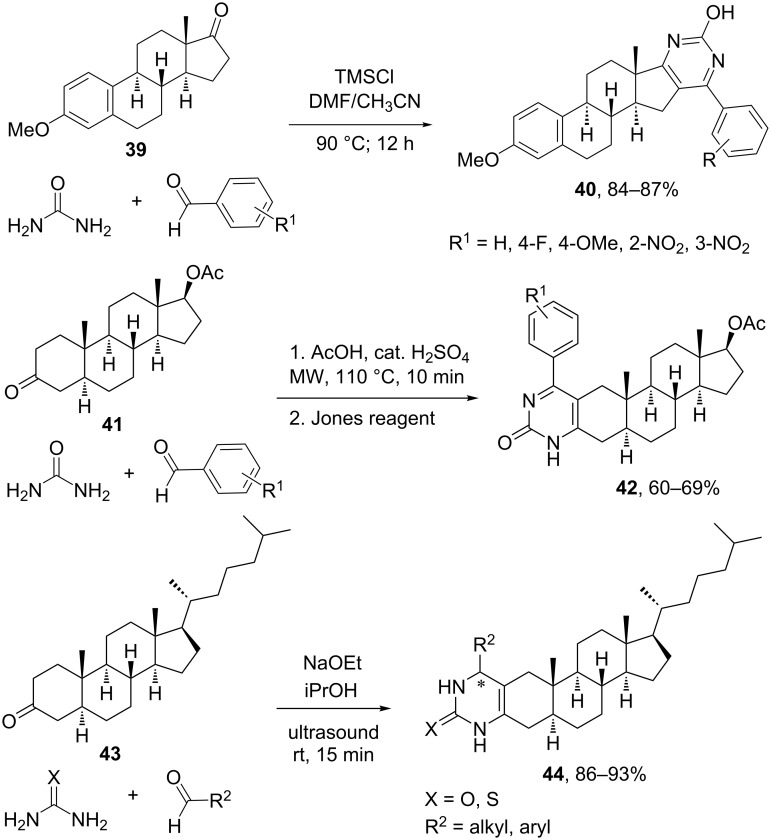
Synthesis of steroid-fused pyrimidines and pyrimidones using the Biginelli-3CR [39,42–43].

In terms of reactants, the reaction seems to work best using aromatic aldehydes with both electron-withdrawing and donating substituents in *ortho*, *meta* and *para*-positions. In addition, not only acetoacetates can be employed, but the reaction can be extended to ketones, thioesters, benzoylacetic esters, acetoacetamides, alkylic or cyclic β-diketones, etc. The urea component has the main structural restrictions, since monosubstituted alkyl ureas work well but thioureas have provided much lower yields. Wang et al. produced a library of steroidal [17,16-*d*]pyrimidine derivatives such as **40** employing a particular extension of the Biginelli reaction based on the use of 17-ketosteroids and chlorotrimethylsilane (TMSCl) as catalyst, which enables the formation of the nucleophilic enolate that attacks either benzaldehyde or its urea imine derivative [39]. Varied ketosteroids were employed, including methylestrone **39**, dehydroepiandrosterone acetate and epiandrosterone acetate, etc., all providing excellent yields of steroidal pyrimidines after 12 h of reaction at 90 °C (Scheme 13). The reaction sequence of this multicomponent protocol comprises the initial formation of the Biginelli product, followed by aromatization under air to furnish the heterocyclic ring fused at positions 16 and 17.

In a recent report, Baji et al. [42] utilized a modified Biginelli-3CR between ketosteroid **41**, urea and different benzaldehyde derivatives for the synthesis of steroid ring A-fused pyrimidinones **42**. In this case, the heterocyclic ring was produced by a second step using the strong oxidizing Jones reagent, because the oxidizing power of sulfuric acid was not enough to achieve oxidation. Interestingly, the use of microwave irradiation instead of classic heating allowed obtaining the Biginelli product in only 10 min, instead of 12 h as required for the synthesis of pyrimidines **40** (Scheme 13).

An alternative setup of Biginelli-3CR with steroids was reported by Boruah and co-workers [43] using ultrasound assistance instead of microwave irradiation or thermal heating. In this case, the reaction between cholestanic ketone **43**, an alkyl or arylaldehyde, and urea or thiourea could be conducted under ultrasound irradiation using sodium ethoxide as catalyst, thus generating steroid ring A-fused 3,4-dihydropyrimidinones and 3,4-dihydropyrimidinthiones **44** in very good yields.

**Scheme 14:**
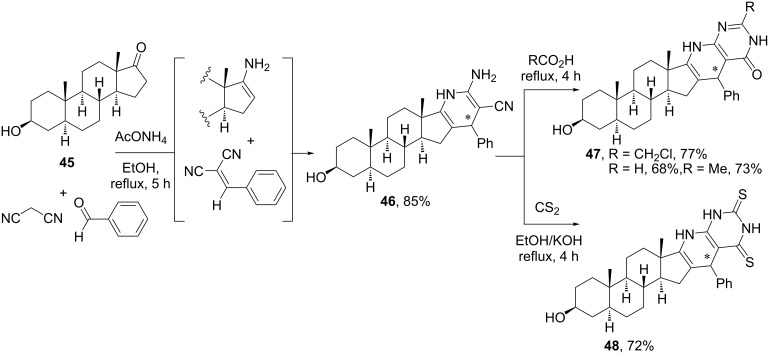
Synthesis of steroidal pyridopyrimidines by a reaction sequence comprising a 4CR followed by a post-MCR cyclization [44].

Mohamed et al. [44] reported a 4CR for the synthesis of pyridopyrimidines fused to ring D of androstanic steroids. Such heterocyclic moieties are of interest because of their pharmacological activity, for example, as anti-inflammatory agents. Employing epiandrosterone and benzaldehyde as oxo components, ammonium acetate and malononitrile as *C*-nucleophile, the authors produced 2-amino-3-cyano-1,4-dihydropyridine **46** fused to the androstane at positions 16 and 17 (Scheme 14). The MCR proceeds by the condensation of malononitrile with benzaldehyde to form the benzylidene malononitrile adduct, and of the ketosteroid with ammonium to form the steroidal enamine, which cyclizes with the benzylidene to furnish the 1,4-dihydropyridine scaffold. As depicted in Scheme 14, compound **46** was later subjected to a variety of post-MCR cyclizations, including the reaction with carboxylic acids to form the fused steroidal pyridopyrimidinones **47** and with carbon disulfide to form pyridopyrimidinedithione **48** in very good yields. Overall, five different components are incorporated in the reaction sequence, whereas acid chlorides, anhydrides, hydrazine, etc., could also be employed in the post-MCR cyclization [44]. The library of steroidal heterocycles was tested against oxidative stress and neuroinflammation due to cerebral injection of lipopolysaccharide endotoxin, with some compounds showing antioxidant and antineuroinflammatory activities. The same group employed this 4CR with cholestan-3-one for the construction of 2-amino-3-cyanodihydropyridine scaffold fused to ring A of the cholestane system [45].

**Scheme 15:**
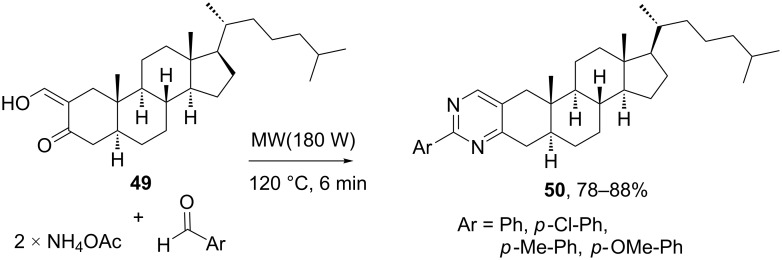
Synthesis of steroid-fused pyrimidines by MCR of 2-hydroxymethylene-3-ketosteroids [46].

Also targeting steroid-fused pyrimidines, Boruah’s group developed a solid-phase MCR between 2-hydroxymethylene-3-ketosteroids, aromatic aldehydes and ammonium acetate [46]. As shown in Scheme 15, the reaction was carried out with steroid **49** and different aldehydes to furnish the set of compounds **50** in good to excellent yields. The proposed mechanism begins with the formation of the β-aminoketoimine by condensation of the steroid with two molecules of ammonia, followed by the reaction with arylaldehyde and intramolecular cyclization to a dihydropyrimidine skeleton, which suffers oxidation to the aromatic heterocyclic ring

Boruah and co-workers extended the same approach to different steroidal starting materials and varied the substituents in the arylaldehyde component to investigate the steric and electronic effects on the yields of the steroid-fused pyrimidine products [43]. Thus, the yield increases when the substituent of the arylaldehyde is in *para*-position, but decrease with the substituent in *ortho*-position, likely due to steric hindrance that limits the cyclization process. In terms of electronic effect, electron-withdrawing groups (e.g., F, Cl, Br, NO_2_) favor the heterocyclization, but electron-donating groups like CH_3_ and OMe does only partially. Both steric and electronic effects can be explained from the reactivity of the diimine intermediate. An electron-withdrawing group promotes the nucleophilic attack of the ketoimine N by increasing the partially positive charge of the imine C. In general, Boruah’s group has been very active in the synthesis of nitrogen heterocycles such as pyrimidines, pyrazolopyrimidines and disubstituted pyridines fused to rings A and D of steroids [47–49].

Thus far, most applications of MCRs in steroid derivatization aimed at producing derivatives for screening their biological and pharmacological properties. A different example is the utilization of MCR-derived steroids as analytical patterns of petroleum samples. Steroids are present as “biomarker” components of petroleum, fused with asphaltenes, which are structurally complex molecules with polycyclic aromatic or partially aromatic hydrocarbons, heteroatoms, alkyl chains and polar functions. As asphaltenes, such steroid-asphaltene derivatives are constituents of the heaviest fractions of petroleum. Understanding the physical characteristics of these derivatives allows their removal from the heavy oil fractions; such compounds are too complex for analysis at a molecular level, so it is important to synthesize analytical standards for their study.

**Scheme 16:**
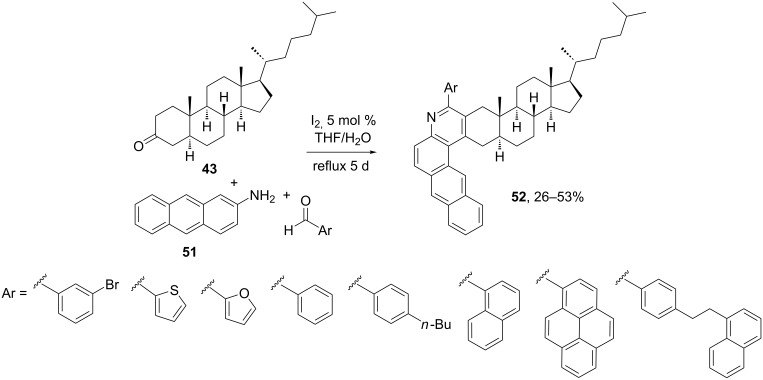
Synthesis of steroid-fused naphthoquinolines by the Kozlov–Wang MCR using ketosteroids [50–51].

In this context, Schulze et al*.* optimized a multicomponent cyclocondensation reaction [50–51] between 5α-cholestan-3-one (**43**), aromatic aldehydes and 2-aminoanthracene (**51**) for the generation of steroidal naphthoquinolines as synthetic asphaltene models for the study of their physical properties. As shown in Scheme 16, the synthetic approach was based on Kozlov–Wang MCR [52], in which an aromatic aldehyde, 2-aminoanthracene and tetrahydropyran-4-one react in the presence of I_2_ as catalyst. The mechanism follows the initial formation of the imine compound between the aldehyde and the arylamine, followed by an imino-Diels–Alder reaction with the enolate generated from the ketosteroid. The reaction is highly regioselective for the enolization of the ketone, thus giving the ring A-fused pyridine at positions 2 and 3. This iodine-promoted MCR tolerates both electron-donating and electron-withdrawing groups on the aromatic aldehyde, thus leading to varied, optically active asphaltene model compounds that could serve as structural mimetics of known components of heavy oils.

Besides the fusion of the steroidal nucleus to heterocycles by MCRs, there is a whole field of research developed by Tietze and co-workers in the 90’s dealing with the construction of heterosteroids, D-homosteroids (steroids with all 6-membered rings) and azasteroids (steroids with a nitrogen atom in their nucleus) [53–54]. This strategy employs domino Knoevenagel/hetero-Diels–Alder procedures for the assembly of such steroid mimics. However, we have decided not to include it in this review because several book chapters and reviews have already covered this chemistry [55–56].

### 4 Conjugation of steroids to carbohydrates and peptides

The conjugation of steroids to other biomolecules such as carbohydrates and peptides represents a valuable strategy for providing new properties to the hydrophobic steroid skeleton. Naturally occurring steroid–sugar conjugates such as saponins have shown to possess physicochemical and biological features different from those of the two separate molecular entities [57–58]. Despite nature does not provide examples of steroid–peptide conjugates, chemists have produced such conjugates to be employed as protease-like artificial enzymes [59] as mimics of the natural cationic peptide antibiotics [60] or as a way to constrain peptide sequences in protein epitope conformations [61]. In this regard, the groups of Rivera and Wessjohann have pioneered the utilization of MCRs for the conjugation of oligosaccharides [62] and peptides [63–64] to steroids, thus producing unique types of steroidal conjugates.

**Scheme 17:**
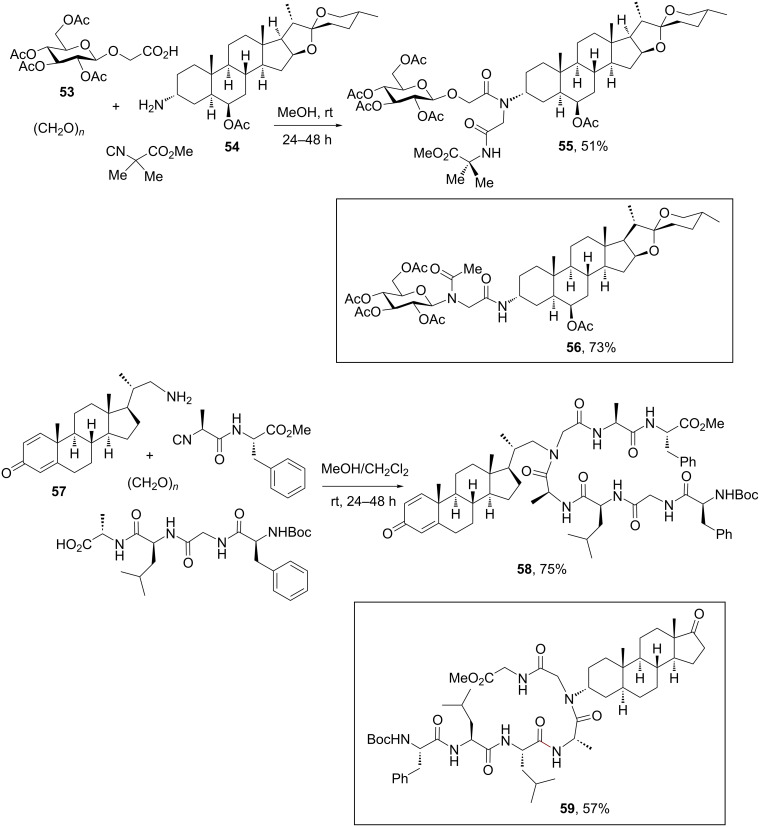
Conjugation of steroids to carbohydrates and peptides by the Ugi-4CR [62–63].

Scheme 17 depicts the strategy toward steroidal conjugates using the Ugi-4CR, which allows accessing a high level of diversity by varying the combinations of the carbohydrate, peptide and steroidal functional groups reacting on the multicomponent conjugation [62–63]. Cytotoxic spirostan saponins were chosen as model compounds for the preparation of Ugi-derived analogues, such as **55** and **56** suitable for biological evaluation [62]. Besides the glucose unit, other trisaccharidic moieties (e.g., β-chacotrioside) could also be conjugated to functionalized spirostanic steroids by Ugi-type MCRs.

In addition, Rivera, Wessjohann and co-workers [63] extended the concept of the Ugi conjugation strategy to a distinctive family of peptide–steroid conjugate having the steroid skeleton or the side chain attached to the peptide backbone as internal *N*-substituent. As shown in Scheme 17, amino steroids could be reacted in MeOH or MeOH/CH_2_Cl_2_ at room temperature with peptide carboxylic acids and isocyanopeptides to furnish peptide–steroid conjugates such as **58** and **59**. Interestingly, the same solution-phase protocol proved to be equally effective for the conjugation of two different steroidal scaffolds [65], but it failed for the ligation of larger peptides to steroids due to the poor solubility of the former ones.

**Scheme 18:**
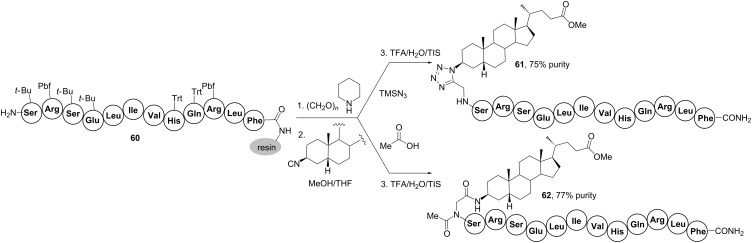
Solid-phase multicomponent conjugation of peptides to steroids by the Ugi-4CR [64].

In this sense, Rivera’s group introduced a solid-phase multicomponent procedure enabling the conjugation of steroids and lipids to peptides longer than 10 amino acid residues [64]. As shown in Scheme 18, the procedure comprised the growth of the antimicrobial peptide sequence **60** followed by the multicomponent incorporation of cholestanic steroids functionalized as isocyanide component. Two related on-resin Ugi-4CRs were employed, i.e., the Ugi-azide reaction based on hydrazoic acid as acid component that furnished peptide-steroid **61** and the classic one using acetic acid as carboxylic acid component, which led to conjugate **62**. These protocols enabled the introduction not only of a steroidal moiety at the *N*-terminus but also of lipid and affinity tags (e.g., biotin) [64]. Importantly, the effective implementation of on-resin Ugi-4CRs paved the way for the subsequent development of multicomponent macrocyclizations permitting the introduction of PEGs, sugars and fluorescent labels at resin-linked peptides [66–67].

**Scheme 19:**
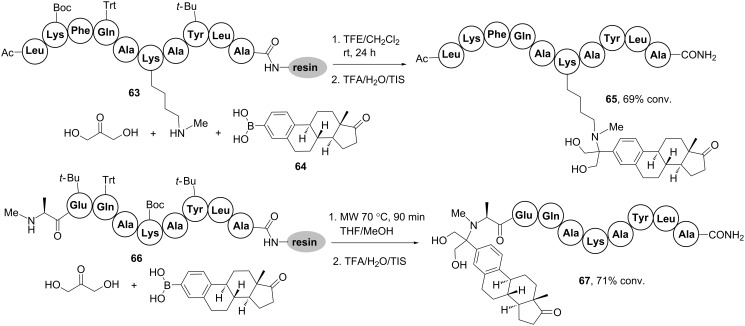
Solid-phase multicomponent conjugation of peptides to steroids by the Petasis-3CR [68].

More recently, Wessjohann and Rivera [68] increased the diversity of MCRs that can be used to ligate peptides to steroids with the development of a novel multicomponent conjugation process based on the Petasis-3CR. The Petasis reaction [69] also known as the borono-Mannich reaction, is a MCR comprising the condensation of an aldehyde or ketone, an amine and an aryl/vinylboronic acid or ester. As illustrated in Scheme 19, the on-resin implementation of this reaction allowed, for the first time, the ligation of oligopeptides to biologically relevant steroids. Thus, resin-linked peptides **63** and **66** could be ligated either by Lys side chains or by the *N*-terminus to the estrone-derived boronic acid **64** using the on-resin Petasis-3CR in the presence of dihydroxyacetone [68]. Peptido-steroids **65** and **67** were obtained in good overall yields after released from the resin using the cocktail TFA/H_2_O/triisopropylsilane (TIS), albeit the conjugation at the *N*-terminus required microwave irradiation due to the bulkier character of the amino component. Besides steroids, the method worked well with varied boronic acids, including those bearing fluorescent labels, lipids and PEG chains, thus providing a new class of biomolecular conjugates showing promise for the future development of peptide pharmaceuticals.

### 5 Multicomponent synthesis of steroidal macrocycles and cages

Since the beginning of this century, the field of macrocycle synthesis has witnessed the emergence of MCRs as effective ring closing procedures, including the cyclization of large scaffolds containing the rigid steroid skeleton. In this regard, Wessjohann can be considered as the pioneer of the synthesis of steroidal macrocycles using MCRs [12–13] including remarkable examples of huge macrocycles formed by the condensation of up to twelve components. Whereas the reports up to 2008 have been included in previous reviews [12–13] this section will cover those reports from 2009 on, with emphasis on multicomponent macrocyclization approaches leading to steroidal cages.

The first report of a MCR-derived steroidal macrocycles was described by Wessjohann et al. in 2005 as part of a synthetic program towards steroid-based supramolecular receptors [28]. There, steroidal diamines and diisocyanides derived from bile acids were employed in a procedure known as MiBs, i.e., multiple multicomponent macrocyclization *i*ncluding bifunctional building blocks. In a series of subsequent reports, Wessjohann’s group exploited the MiBs strategy based on the Ugi-4CR for the assembly of topologically diverse steroidal macrocycles incorporating a variety of (seco)steroid skeletons [70–72].

**Scheme 20:**
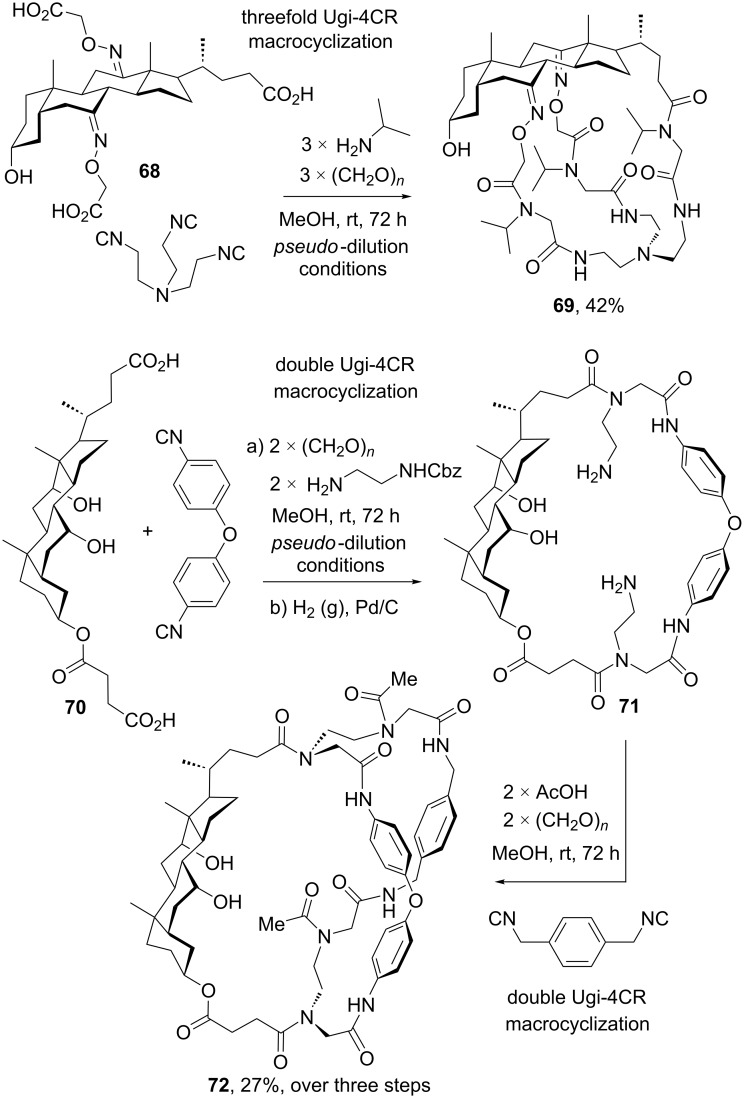
Synthesis of steroidal macrobicycles (cages) by multiple multicomponent macrocyclizations based on the Ugi-4CR [73,75].

Probably the highest level of macrocycle complexity achieved in one-pot can be found in the multicomponent synthesis of steroidal cages multiple multicomponent macrocyclization [73]. As shown in Scheme 20, Rivera and Wessjohann expanded the MiBs concept to the development of a threefold Ugi-4CR-based macrocyclization between cholanic tricarboxlic acid **68** and an aliphatic triisocyanide in the presence of three equivalents each of paraformaldehyde and isoprolylamine to furnish steroidal cage **69** in good yield. This multicomponent macrocyclization approach was implemented by setting up *pseudo*-dilution conditions [74], i.e., the simultaneous slow addition of two trifunctional components with syringe pumps to a stirring solution of the pre-formed imine (3 equiv), which avoids formation of higher oligomers. Remarkably, such procedure was undertaken with formation of twelve covalent bonds and the incorporation of eight components in one pot. A few years later, the same authors described the implementation of a sequential MiBs strategy for the construction on hybrid macromulticycles including a steroidal skeleton as one of the tethers [75]. Scheme 20 illustrates one of the examples reported by Wessjohann’s group, in which cholanic dicarboxylic acid **70** was subjected to an initial macrocyclization based on two Ugi-4CRs with a biaryl ether diisocyanide and two equivalents of paraformaldehyde and a monoprotected diamine. After the initial macrocyclization, steroidal macrocycle **71** was deprotected by removal of the Cbz groups and subsequently submitted to a second multicomponent macrocyclization protocol – in this case serving as diamino component – to render macrobicycle **72** in moderate yield over three steps. Despite the process was not conducted in one pot, this simple synthetic setup was employed to produce a set of very complex steroidal macrobicycles suitable for molecular and ion pair encapsulation [75].

**Scheme 21:**
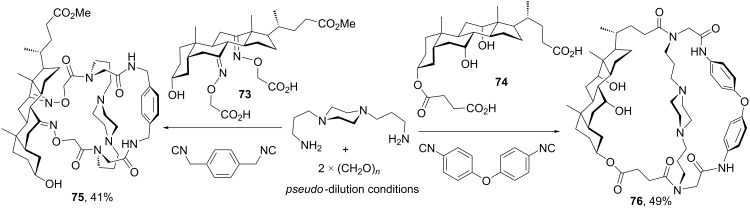
One-pot synthesis of steroidal cages by double Ugi-4CR-based macrocyclizations [76].

Aiming at producing a more efficient and straightforward method toward macrobicycles, Wessjohann and Rivera [76] implemented a double Ugi-4CR-based macrocyclization comprising the one-pot assembly of three different bifunctional building blocks, one of them of steroidal nature. Differently from any other MiBs approach, this procedure focuses on constructing the macrobicycle connectivity by the bridgeheads instead of the tethers, which are brought together by the two Ugi-4CRs. As shown in Scheme 21, this method allows the assembly of hybrid steroidal cages with the incorporation of three bifunctional components. Thus, cholanic dicarboxylic acids **73** and **74** were macrocyclized with a paraformaldehyde-derived diimine and two different aryl diisocyanides to furnish steroidal cages **75** and **76** in good yields considering the structural complexity created in a single synthetic operation. It is worth mentioning that due to the rigid nature of the steroidal and aryl components, a flexible diamine was required to facilitate the macrobicycle ring closure. As before, *pseudo*-dilution conditions were employed by the simultaneous slow addition of the three bifunctional components.

## Conclusion

We have demonstrated that MCRs are powerful tools for the derivatization of steroids, including the formation of steroidal heterocycles and macrocycles and the conjugation to other biomolecular components. Various well-known MCRs such as the Ugi, Passerini, Biginelli and Petasis reactions have proven effective in the installation of skeletal diversity attached to the steroid ring system, while many other modern methods have also shown success using steroidal as one of their components. In addition, we showed that the synthesis of steroid–peptide and steroid–carbohydrate conjugates is feasible by means of both solution and solid-phase multicomponent methodologies, which opens a venue of possibilities for the production of more complex biomolecular conjugates. Nonetheless, there are still many MCRs that have not been implemented using steroidal substrates, therefore, there is still much to be done for an effective exploitation of the MCR potential in the rapid diversification of steroidal products for screening of their pharmaceutical and biological properties. We expect this review serves as inspiration for the MCR community to further explore the scope of steroids in the development of new reactions and methods.
